# Supplementary material for: Complex‐centric proteome profiling by SEC‐SWATH‐MS
Source: Mol Syst Biol. 2019 Jan 14;15(1):e8438. doi: 10.15252/msb.20188438 (PMC6346213; doi:10.15252/msb.20188438)
Supplement: Supplementary file 8 — Dataset EV7 [file MSB-15-e8438-s008.zip › feature_plots_string/P19878.pdf]

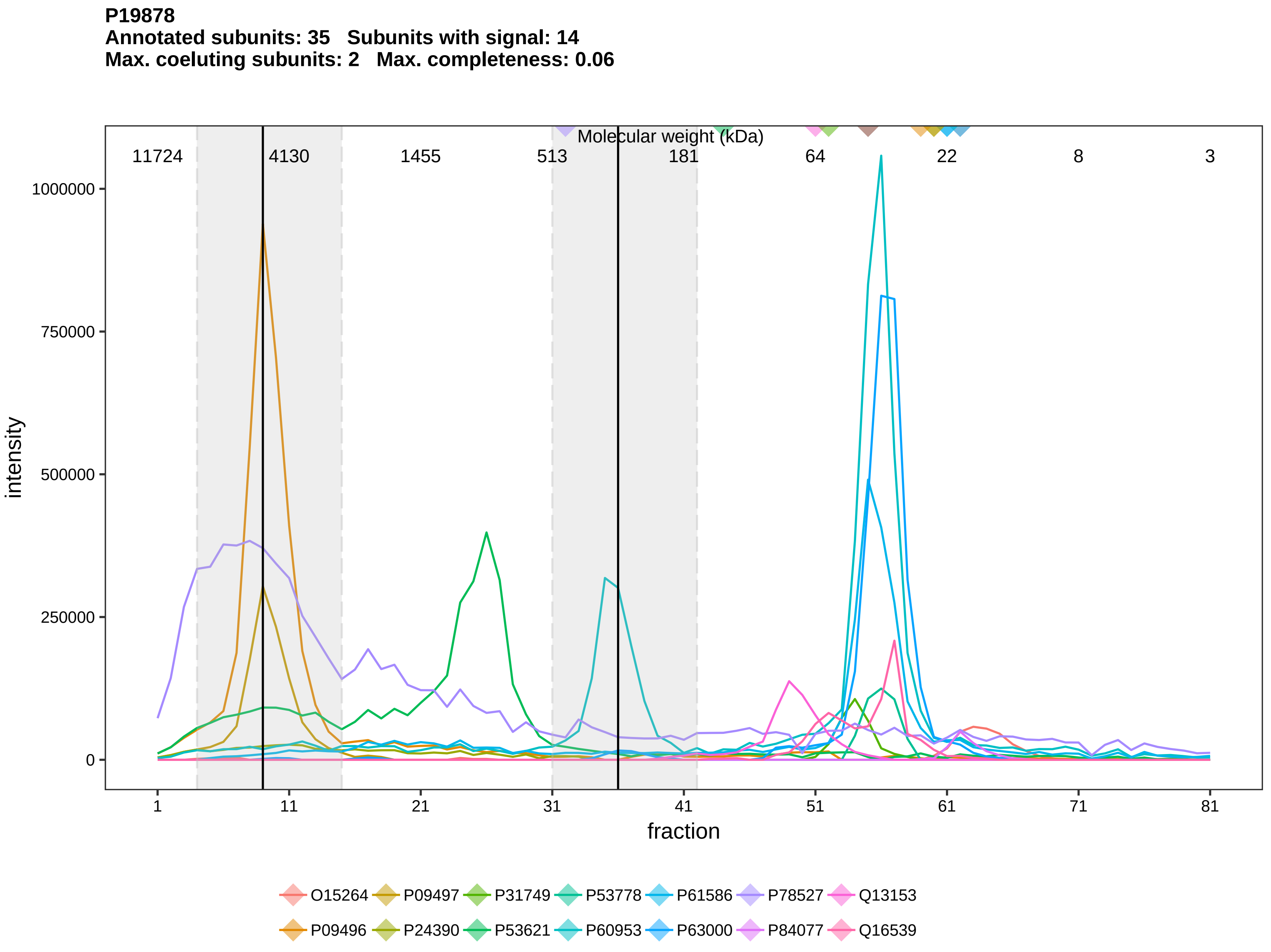

P19878
Annotated subunits: 35 Subunits with signal: 14
Max. coeluting subunits: 2 Max. completeness: 0.06
Molecular weight (kDa)
11724
4130
1455
513
181
64
22
8
3
intensity
1000000
750000
500000
250000
0
1
11
21
31
41
51
61
71
81
fraction
O15264
P09497
P31749
P53778
P61586
P78527
Q13153
P09496
P24390
P53621
P60953
P63000
P84077
Q16539
